# Low lateral inclination angle, high sulcus angle, high trochlear height and patella alta are risk factors for first lateral patellar dislocation and complete MPFL rupture, comparative study

**DOI:** 10.1002/jeo2.70213

**Published:** 2025-03-22

**Authors:** Serhat Akcaalan, Ismail Duran, Abdurrahim Kavaklilar, Fatih Beser, Ceyhun Caglar, Mahmut Ugurlu

**Affiliations:** ^1^ Department of Orthopedics and Traumatology Ankara City Hospital Ankara Turkey; ^2^ Department of Orthopedics and Traumatology Ankara Yıldırım Beyazıt University Ankara Turkey

**Keywords:** instability, morphometry, MPFL, patellar, rupture

## Abstract

**Purpose:**

To identify risk factors for complete medial patello‐femoral ligament (MPFL) rupture after first lateral patellar dislocation (LPD) and to develop a model to predict the risk of rupture.

**Methods:**

Patients who presented with first LPD between February 2019 and June 2024 and were diagnosed with complete MPFL rupture on magnetic resonance imaging (MRI) were retrospectively reviewed. Patients with normal MRI findings in a 1:1 ratio were selected as the control group by computer‐assisted randomisation.All patients in both groups were asked to perform MRI on, tibial tuberosity–trochlear groove (TT–TG) distance, lateral trochlear inclination (LTI) angle, sulcus angle (SA), medial femoral condyle height (MFCH), lateral femoral condyle height (LFCH), trochlear height (TH), patellotrochlear index (PTI), Koshino–Sugimoto Index (KSI), Caton–Deschamps Index (CDI) and Insall–Salvati Index (ISI) were measured and recorded. All measurements were made by two different orthopaedists and intra‐observer reliability was evaluated. The measurements between the groups were compared statistically.

**Result:**

A total of 98 patients, including 49 patients with complete MPFL rupture (study group) and 49 patients in the control group, were included in the study. Thirty of the patients in both groups were males and 19 were females. Mean age was 23.55 years in the study group and 24.29 years in the control group (*p* = 0.447). Satisfactory ICC scores were obtained in all measurements. LTI was lower in the study group than in the control group (*p* = 0.002), while SA was higher in the study group than in the control group. Both CDI and ISI were statistically significantly higher in the study group compared to the control group (*p* = 0.002, *p* = 0.003). The probability of predicting the risk of complete MPFL rupture of the risk analysis model created with radiological risk factors for complete MPFL rupture was 70.4%.

**Conclusion:**

LTI, SA, TH and patella alta are risk factors for complete MPFL rupture after first LPD. Risk analysis of complete MPFL rupture after first dislocation can be successfully performed with MRI findings. This risk analysis can be used to predict the risk of developing complete MPFL after primary LPD, especially in risky patient groups, and can be used in a simple way to decide which patients will receive a preventive programme without the need for additional examination.

**Level of Evidence:**

Level III, case–control study.

**Abbreviations**
CDI: Caton–Deschamps Index
ICC: intraclass correlation coefficient
ISI: Insall–Salvati Index
KSI: Koshino–Sugimoto Index
LFCH: lateral femoral condyler height
LPD: lateral patellar dislocation
LTI: lateral trochlear inclination
MFCH: medial femoral condyler height
MPFL: medial patello femoral ligament
MRI: magnetic resonance imaging
OR: odds ratio
PTI: Patello‐Trochlear Index
SA: sulcus angle
SPSS: Statistical Package for the Social Sciences
TH: trochlear height
TT–TG: tibial tuberosity–trochlear groove

## INTRODUCTION

Patellar instability (PI) is a serious orthopaedic problem that can be seen in both athletes and non‐athletes, causing pain, dysfunction, and absence from sports [19]. It is most common in the 10–16 age group, with an incidence of 5.8 per 100,000 people [35]. The cause of PI is multifaceted.

In the development of lateral patellar dislocation (LPD), demographic risk factors such as age, gender and activity level have an effect as well as anatomical risk factors [13]. Research on anatomical risk factors has mostly focused on abnormal structures in the patellofemoral joint structure [23, 31]. Lateralisation of the tibial tubercle, trochlear dysplasia and patella alta are among the anatomical risk factors contributing to the development of instability [8, 18, 35].

If insufficient medial patello femoral ligament (MPFL) occurs after LPD, recurrent patella dislocations are likely to develop [28]. Therefore, MPFL reconstruction after LPD is the most common surgical approach for patellar stabilisation [17, 25]. However, MPFL reconstruction alone in patients with anatomical risk factors is associated with poor clinical outcome and re‐dislocation [11, 32]. Therefore, it is very important to reveal the relationship between patellar dislocation and bone morphometry. In addition to its effect on surgical decision making, it is also important to clearly demonstrate the relationship between bone morphometry and patellar dislocation for the development and implementation of preventive measures. Similarly, preventive studies can be planned by clearly identifying the anatomical risk factors for patella dislocation.

This study is a magnetic resonance imaging (MRI)‐based study planned to identify morphometric risk factors in patients with MPFL rupture after patellar dislocation. It was planned to identify risk factors for MPFL rupture after the first LPD and to develop a radiologic measurement‐based model for MPFL rupture. The authors hypothesise that MRI findings can be used to create a risk analysis to guide treatment planning.

## MATERIALS AND METHODS

The study is a case–control study with a retrospective design. Approval for the study was obtained from our hospital's clinical studies ethics committee (1‐24‐391). Patients with complete MPFL rupture on MRI who were admitted to our hospital's orthopedics and traumatology clinic due to initial patella dislocation were included in the study. Exclusion criteria from the study were that the patient's MRI images were not of sufficient quality to make measurements, generalised ligamentous laxity (Marfan and Ehler‐Danlos etc.), previous knee ligament injury Those with a history of neurovascular injury in the extremity examined, those with a history of previous knee surgery (knee arthroscopy, tibial tubercle osteotomy, MPFL reconstruction etc.), patients with osteoarthritis findings in MRI and X‐ray (Kellgren–Lawrence grade 2 and severe), BMI > 30, elite athlete, spontaneous LPD and patients with a history of knee circumference fractures without surgery were determined. Control group images were selected from an equal num ber of patients who met the inclusion criteria, with no pathological MRI findings (e.g., meniscus tear, MPFL rupture, anterior/posterior cruciate ligament rupture, collateral ligament damage), and no history of LPD.

**Figure 1:**
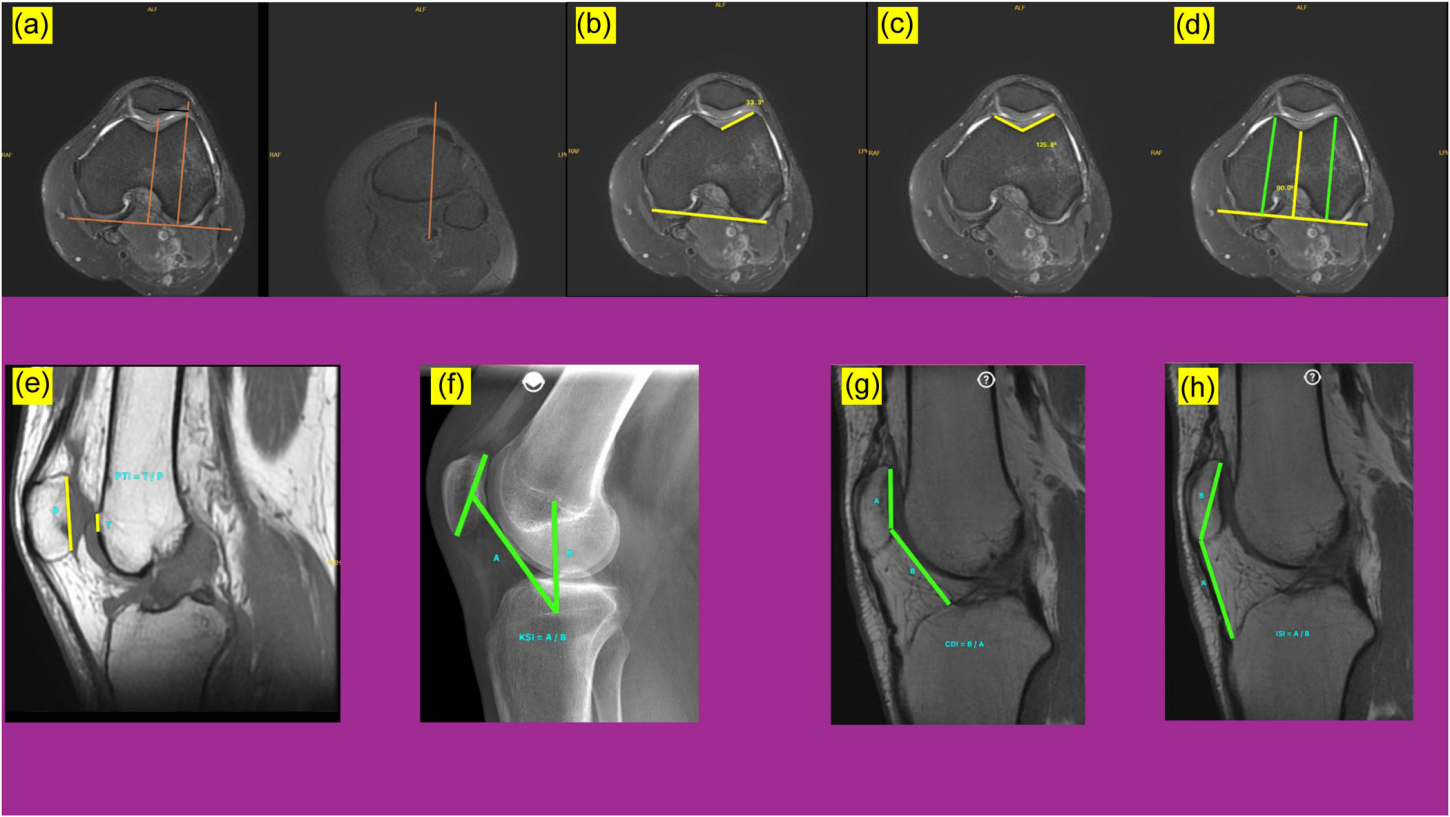
TT–TG distance (a), Lateral Trochlear Index (b), sulcus angle (c), medial lateral femoral condyler height, lateral femoral condyler height and trochlear height (d), Patellotrochlear Index (e), Koshino–Sugimoto Indeks (f), Caton–Deschamps Index (g), Insall–Salvati Index (h).

The age and gender information of all patients in both the rupture group and the control group were recorded. All patients underwent the following measurements on MRI. Of the specified measurements, only the Koshino–Sugimoto Index (KSI) was performed on the lateral knee radiograph. All measurements were performed by two different orthopaedists who were not authorised to access the demographic data of the patients. The average of both measurements was used for statistical analysis. Reliability and validity analysis of the measurements were performed. All measurements performed on MRI are shown in Figure 1.

### Radiological measurements

1. **Tibial tuberosity–trochlear groove (TT–TG) distance:** [26] It was measured as the distance in millimetres (mm) between the most anterior part of the tibial tuberosity and the central part of the trochlear groove.
2. **Lateral trochlear inclination (LTI)**: [3] It is the angle between the line drawn parallel to the lateral trochlear subchondral bone on the axial MRI image and the line joining the posterior femoral condyles on the same MRI image.
3. **Sulcus angle (SA)**: [27] The sulcus angle was measured as the angle between two lines tangent to the medial and lateral trochlear facets.
4. **Medial femoral condylar height (MFCH)**: [16] In the axial MRI image, a line was drawn to the most posterior part of the femur, in this sequence, a line was drawn from the most anterior part of the medial femoral condyle perpendicular to the posterior line. This distance was recorded in mm and MFCH was calculated.
5. **Lateral femoral condylar height (LFCH)**: [16] In the axial MRI image, a line was drawn to the most posterior part of the femur, in this sequence, a perpendicular line was drawn from the most anterior part of the lateral femoral condyle to the posterior line. This distance was recorded in mm and MFCH was calculated.
6. **Trochlear height (TH)**: [16] In the axial MRI image, a line was drawn to the most posterior part of the femur, in this sequence, a perpendicular line was drawn from the central point of the trochlea to the posterior line. This distance was recorded in mm and MFCH was calculated.
7. **Patellotrochlear Index (PTI):** [1] PTI: (A/B), A: Distance between the most superior aspect of the trochlear articular surface to the most inferior aspect of the patellar articular cartilage. B: Distance between the most superior aspect of articular cartilage to the most inferior aspect of articular cartilage of the patella.
8. **Koshino–Sugimoto Index (KSI)**: [15] The KSI method calculates the index as the ratio of the distance of the distance between the centre of the patella and the centre of the proximal tibial physis to the distance between the centre of the femoraldistal physis and the centre of the proximal tibial physis.
9. **Caton–Deschamps Index (CDI)**: [4] The CD method calculates the index as the ratio of the distance from the inferior point of the patellar articular surface to the anterior most point of the tibial plateau to the length of the patellar articular surface.
10. **Insall–Salvati Index (ISI**): [12] ISI method calculates the index as theratio of the distance from the inferior point of the patellar articular surface to the patellar tendon attachment on the tibia to the length of the patellar articular surface.

G.Power version 3.1.9.7 was used to determine the required sample size. Fifteen patients from the control and study groups were included in the analysis for the study, the most significant relationship was found in the distance between the sulcus height. The total required sample size was calculated as 97 in the analysis with an odds ratio of 1.93, a significance level of 0.05 and a test power of 0.8.

The morphometric measurements were performed by two orthopaedists using single blinding method and the agreement between the results was analysed by intraclass coefficient (ICC). The authors referenced the values in the study by Koo et al. as acceptable values for ICC [14]. ICC scores of the measurements are shown in Table 1.

**Table 1:** Intraclass coefficient (ICC) scores of all measurements specified in Section 2.

Parameters	ICC score
Tibial tuberosity–trochlear groove (TT–TG) distance	0.903
Lateral trochlear inclination	0.892
Sulcus angle	0.881
Medial femoral condylar height	0.912
Lateral femoral condylar height	0.921
Trochlear height	0.902
Patellotrochlear Index	0.912
Koshino–Sugimoto Indeks	0.887
Caton–Deschamps Index	0.913
Insall–Salvati Index	0.914

### Statistical analysis

Statistical analysis of the data obtained in this study was performed using SPSS 26.0 (Statistical Package for the Social Sciences) software. Descriptive statistics were calculated to evaluate the demographic data and morphometric parameters associated with MPFL tear. Mean and standard deviation values were presented for continuous variables, while categorical variables were expressed as frequency and percentage distributions. In analysing the differences between two groups, an independent sample *t*‐test was used for normally distributed data and Mann–Whitney *U* test was used for non‐normally distributed data. Logistic regression analysis was performed to determine the risk factors for MPFL tear and the results were evaluated by calculating odds ratios of significant variables. The significance level was accepted as *p* < 0.05 in all analyses. In this study, logistic regression analysis was performed using the backward elimination method to determine the variables affecting the development of MPFL rupture.

## RESULTS

A total of 98 patients were included in the study.49 patients were in the study group (patients with MPFL rupture after LPD), and 49 patients were in the control group. The distribution of males and females was the same in both groups: 30 males and 19 females. The mean age of the patients in the study group was 23.55 ± 5.59, while the mean age of the patients in the control group was 24.29 ± 3.76. There was no statistically significant difference between the groups in terms of age (*p* = 0.447).

The data of both the study and control groups for the parameters whose measurement methods were described in detail above are shown in Table 2.

**Table 2:** *p* values after comparison with the mean and standard deviations for both groups of measurements specified in Section 2.

Parameters	Study group *n* = 49	Control group *n* = 49	*p* value
TT–TG distance (mm)	17.2 ± 4.4	16.0 ± 4.8	0.21
Lateral trochlear inclination	22.6 ± 4.8	25.7 ± 5.0	0.002*
Sulcus angle	133.7 ± 7.1	129.3 ± 6.9	0.002*
Medial femoral condylar height (mm)	59.3 ± 4.6	60.1 ± 4.8	0.434
Lateral femoral condylar height (mm)	60.6 ± 4.7	60.7 ± 5.3	0.971
Trochlear height (mm)	48.1 ± 5.3	44.3 ± 5.5	0.001*
Patellotrochlear Index	0.5 ± 0.1	0.5 ± 0.1	0.084
Koshino–Sugimoto Index	1.3 ± 0.1	1.2 ± 0.1	0.392
Caton–Deschamps Index	1.3 ± 0.2	1.1 ± 0.2	0.001*
Insall–Salvati Index	1.2 ± 0.2	1.0 ± 0.2	0.003*

Abbreviation: TT–TG, Tibial tuberosity–trochlear groove.

In this study, four different measurement techniques were used to determine the patellar height. Of these, a moderate agreement was found between CDI and ISI, and this agreement was statistically significant (kappa = 0.429, *p* = 0.000). Very poor agreement was observed between CDI and KSI and PTI, which were not statistically significant (kappa = 0.112, *p* = 0.150; kappa = 0.02, *p* = 0.504, respectively).

As a result, LTI, MFCH, TH and CDI results categorised according to patella position (patella alta, normal and patella baja) were found to have significant relationships in the model. LTI has a negative coefficient and each unit increase in this index decreases the risk of MPFL rupture by 13.1% (Exp(*B*) = 0.869, 95% *p* = 0.013). Similarly, MFCH also shows a negative association, with each unit increase in this value reducing the risk of MPFL rupture by 18.9% (Exp(*B*) = 0.811, 95% *p* = 0.002). On the other hand, TH had a positive association, with each unit increase in TH increasing the risk of developing MPFL rupture by 28% (Exp(*B*) = 1.280, *p* = 0.000). CDI was found to be a significant predictor and according to the model result, the risk of MPFL rupture increased approximately 2.65 times (Exp(*B*) = 2.646 *p* = 0.067) in individuals with an patella alta compared to those with a normal patella position. The data of the parameters used in the model are shown in Table 3.

**Table 3:** Data regarding the parameters in the model created to estimate the risk of complete MPFL rupture after primary LPD.

	*B*	S.E.	Wald	*df*	Sig.	Exp (*B*)
*LTI*	−0.140	0.056	6.187	1	**0.013**	**0.869**
*MFCH*	−0.210	0.069	9.316	1	**0.002**	**0.811**
*TH*	0.247	0.064	14.906	1	**0.000**	**1.280**
*CDI*	0.973	0.532	3.343	1	**0.067**	**2.645**
*Constant*	4.771	3.490	1.869	1	**0.172**	**117.990**

*Note*: Nagelke *R* = 0.397, Omnibus chi‐square = 35.493, Hosmer–Lemeshov = 0.768.

Abbreviations: CDI, Caton–Deschamps Index; *df*, degrees of freedom; LPD, lateral patellar dislocation; LTI, lateral trochlear inclination; MFCH, medial femoral condyler height; MPFL, medial patello femoral ligament; S.E., standard error; TH, trochlear height.

According to the classification table, the power of the model to predict MPFL rupture was calculated as 70.4%. This accuracy rate indicates that the model has a reasonable level of success in distinguishing between intact and MPFL‐ruptured individuals. The model correctly classified 71.4% of intact individuals and 69.4% of individuals with MPFL rupture. These results indicate that the model has a moderate predictive power in assessing the risk of MPFL rupture. The results are shown in Table 4.

**Table 4:** Data showing the predictive power of the model constructed to estimate the risk of complete MPFL rupture after primary LPD.

Observed		Predictionary		
		MPFL		Percentage correct
		Intact	Rupture	
*MPFL*	*Intact*	35	14	**71.4**
	*Rupture*	15	34	**69.4**
*Overall percentage*				**70.4**

*Note*: The cut value is 0.500.

Abbreviations: LPD, lateral patellar dislocation; MPFL, medial patello femoral ligament.

## DISCUSSION

The most important finding of this study is that patellar height was statistically significantly higher, LTI was increased, SA was increased and TH was higher in the MPFL‐ruptured group compared to the MPFL intact group.

Trochlear dysplasia is one of the most important components contributing to recurrent PI.LTI is a defined measure of trochlear morphologic features [3]. LTI has previously been used in different studies to define trochlear dysplasia [5, 29]. LTI was determined to be the most highly rated measurement technique by the experts among all measurements defining trochlear dysplasia [21]. In their original study defining LTI, Carrillon et al. [3] stated that the cut‐off value of LTI in defining trochlear dysplasia was 11° and that values below 11° were associated with trochlear dysplasia. In another study, the LTI value for normal knees was determined as 17°, but they stated that a cut‐off value of 11° could be used appropriately for dysplasia [21]. In our study, LTI was found to be statistically significantly lower in the group with complete MPFL rupture compared to the control group. However, the values obtained in our study were found to be in a much different range than the values in the literature. The mean LTI value was 22.6° in the MPFL‐ruptured group and 25.7° in the control group. Our study supports the information that LTI is lower in patients with complete MPFL rupture compared to the control group, but it does not support the cut‐off value of LTI to reveal dysplasia and its value in normal knees. It is known that LTI is one of the best measurement techniques to define trochlear dysplasia, but it is one of the secondary results of our study that large participatory studies are needed to determine cut‐off values.

Sulcus angle is one of the measurements used to define trochlear morphology and has been used in different studies to define trochlear dysplasia [9, 30]. In their systematic review, Saccomanno et al. stated that the sulcus angle is one of the most investigated parameters used to define trochlear dysplasia [24]. Gobbi et al. [10] evaluated 142 patients with PI and showed that the trochlear sulcus angle was ≥153 in patients with PI. Different sulcus angle cut‐off values have been reported to define trochlear dysplasia in patients with PI [24]. In our study, we found that the sulcus angle was significantly higher in patients with complete MPFL rupture compared to the control group (*p* = 0.002). However, as in LTI, it should be noted that there are many different numerical values in the literature but there is no clear consensus. Another parameter used to define trochlear dysplasia is trochlear height [9]. Pruneski et al. [23] showed that trochlear height increased in patients with PI in both male and female gender. In parallel with the literature, we found statistically higher THI in the patient group with complete MPFL rupture.

Patella alta is a well‐known risk factor for PI that has been widely studied in the literature [6]. Patella alta creates a risk for PI by causing the patella not to enter the trochlear groove at higher degrees of knee flexion [2]. Many techniques have been described in the literature to define patellar height, but there is no gold standard measurement technique. The most commonly used techniques used to measure patellar height in the literature are ISI and CDI [33]. In their meta‐analysis, White et al. [33] evaluated patellar height as a sub‐heading; they reported that ISI was statistically higher in the PI group compared to the control group, but there was no statistically significant difference between the two groups in CDI. In another study, it was shown that both high CDI and high ISI, that is, greater than 1.2, are risk factors for PI [20]. In our study, we found that both CDI and ISI were higher in the MPFL rupture group. In the group with MPFL rupture, CDI was 1.3 and ISI was 1.2. Our study showed that patella alta is a risk factor for MPFL rupture after first LPD.

There are many measurement techniques in the literature to determine patellar height, but there is no gold standard measurement technique among them. In our study, we evaluated 4 different patellar height measurements. We found only CDI and ISI to be associated with complete MPFL rupture. When we looked at the agreement between each other, we found a moderate agreement only between CDI and ISI. Although the reliability and validity rates of all 4 methods are high, we found that methods other than CDI and ISI are insufficient to support the risk analysis of complete MPFL rupture. The International Patellofemoral Study Group also uses the CDI as the preferred method for measuring patellar height [17]. In addition, in the modified Delphi consensus statement on PI published in 2023, it was stated that CDI is the preferred method for patellar height measurement [7]. Our study supports these recommendations because the statistical power of CDI is higher than ISI.

Predicting whether re‐dislocation will develop after LPD dislocation is very important both for treatment selection and for returning to sports activities and preventive studies.The rate of re‐dislocation after LPD varies between 8.6% and 88% depending on individual patient factors [22]. Wierer et al. tried to calculate the probability of PI with their study [34]. In this study, they found that young age and trochlear dysplasia were the main risk factors for early recurrent LPD and the accuracy of the calculator they created was 79%. In our study, we found that the rate of prediction of complete MPFL rupture was 70.4% in our model that we created with only radiological data. This model can be improved by adding demographic data of the patients and can be used both for treatment selection and for risk analysis to develop preventive health measures.

First of all, the retrospective design of the study naturally constitutes a limitation. The fact that the radiological measurements were performed in 2D and the measurements were not supported by any artificial intelligence or computer programme is another limitation of the study. This risk analysis includes only radiographic measurements, demographic and clinical variables that could increase accuracy are not included. Other limitations of the study are that external validation of this created model is not performed and the accuracy of the created model is not compared with other models in the literature.

## CONCLUSION

In conclusion, low lateral trochlear inclination angle, high sulcus angle, high trochlear height and patella alta, which indicate trochlear dysplasia, are predisposing factors for complete MPFL rupture after LPD. The model we created using these risk factors can predict the probability of complete MPFL rupture after first LPD with an accuracy of 70.4%. This risk analysis can be used to predict the risk of developing complete MPFL after primary LPD, especially in risky patient groups, and can be used in a simple way to decide which patients will receive a preventive programme without the need for additional examination.

## AUTHOR CONTRIBUTIONS

Serhat Akcaalan and Fatih Beser developed the theoretical formalism, performed the analytic calculations and performed the numerical simulations. Ismail Duran and Abdurrahim Kavaklilar perform measurement. Both Serhat Akcaalan and Ceyhun Caglar authors contributed to the final version of the manuscript. Mahmut Ugurlu supervised the project.

## CONFLICT OF INTEREST STATEMENT

The authors declare no conflicts of interest.

## ETHICS STATEMENT

There is an ethical approval. It will be uploaded as an additional file. The study is a retrospective screening study.

## Data Availability

All data generated or analysed during this study are included in this published article.
